# Unusual Cause of Small Bowel Obstruction Caused by a Prune Pit

**DOI:** 10.5334/jbr-btr.838

**Published:** 2015-09-15

**Authors:** B. Geerts, T. Verstraeten, K. Vanslambrouck, S. Vandenhaute, B. Dillemans, K. Coenegrachts

**Affiliations:** 1 Department of Radiology, AZ Sint-Jan Brugge-Oostende AV, Bruges, Belgium; 2 Department of Surgery, AZ Sint-Jan Brugge-Oostende AV, Bruges, Belgium

An 86-year-old woman came to our emergency department with a history of nausea and diffuse abdominal pain during the last 24 hours. Since then, she had absence of flatus and stool. At the age of 47, she underwent an appendectomy.

Clinical examination revealed a distended and hypertympanic abdomen without bowel peristalsis. Laboratory examination showed slightly increased inflammatory parameters.

An upright plain film of the abdomen (Fig. A) revealed multiple air-fluid levels and distended bowel loops. There was no pneumoperitoneum. An aspecific opacity was seen in the pelvis. There is a well-defined translucent supradiaphragmatic area compatible with a sliding hiatus hernia. Ultrasound was performed and revealed dilated small bowel loops. The gallbladder was hydroptic but there was no wall thickening. A CT was performed with IV and retrograde contrast agent opacification, the axial and coronal reformatted images (Figs. B and C) of the abdomen showed a well-defined dense double-layered intraluminal structure in the preterminal ileum and multiple distended bowel loops.

**Figures A–C:**
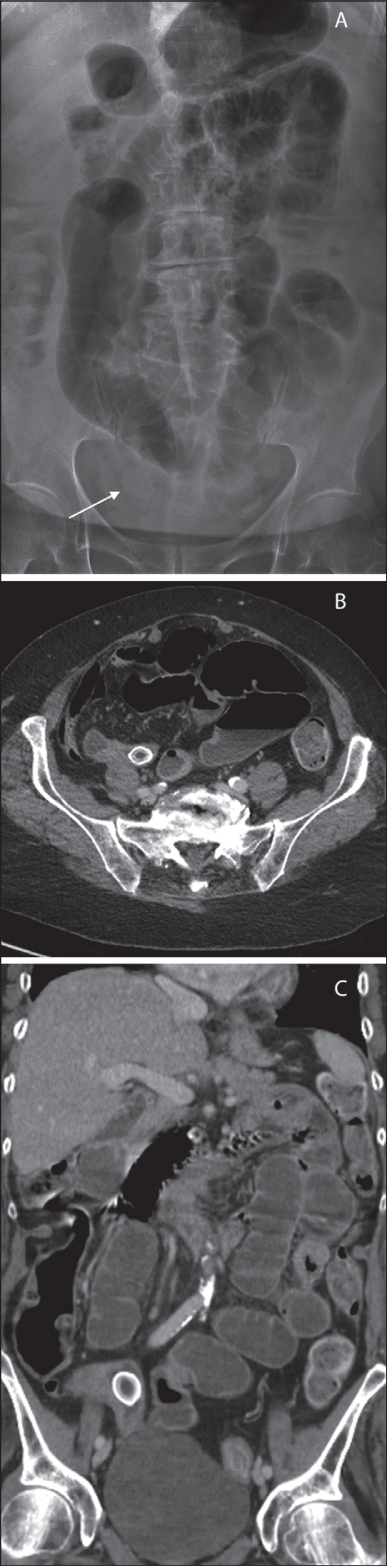


A small bowel obstruction caused by a corpus alienum was diagnosed on the basis of radiograph, ultrasound and CT findings.

The patient underwent a laparotomy which revealed a prune pit and multiple adhesions at the site of obstruction. The adhesions were possibly due to the appendectomy at the age of 47.

## Comment

Small bowel obstruction (SBO) is a common condition that is secondary to mechanical or functional obstruction in the small bowel, preventing normal transit. SBO can have multiple causes and the diagnosis is based on a combination of patient history, physical examination, clinical background and radiologic findings. To date, the existing literature describes no case of small bowel obstruction caused by a prune pit. In this case, the adhesions at the preterminal ileum, possibly due to the appendectomy, could cause restriction of small bowel mobility. This might be a contributing factor to the particular location of the obstruction. Dried apricots are sometimes seen as a cause of small bowel obstruction, frequently in elderly people. Gall stone ileus was excluded in this case since there was no detection of free air within the bile ducts, nor pericholecystic fluid or other signs of cholecystitis. Other intraluminal causes of small bowel obstruction are bezoars, parasites and enteroliths.

## Competing Interests

The authors declare that they have no competing interests.
